# Loss of PIK3CA Allows *In Vitro* Growth but Not *In Vivo* Progression of KRAS Mutant Lung Adenocarcinoma in a Syngeneic Orthotopic Implantation Model

**DOI:** 10.3390/cells15060506

**Published:** 2026-03-12

**Authors:** Abigail L. Booth, Giuseppe Caso, Barbara Rosati, Ya-Ping Jiang, Wei-Xing Zong, Richard Z. Lin, Harold Bien

**Affiliations:** 1 Department of Physiology & Biophysics, Stony Brook University, Stony Brook, NY 11794, USA; abigail.booth@stonybrook.edu (A.L.B.); richard.lin@stonybrook.edu (R.Z.L.); 2 Molecular and Cellular Biology Graduate Program, Stony Brook University, Stony Brook, NY 11794, USA; 3 Stony Brook University Single Cell and Spatial Multiomics Facility, Stony Brook University, Stony Brook, NY 11794, USA; 4 Department of Chemical Biology, Ernest Mario School of Pharmacy, Rutgers-The State University of New Jersey, Piscataway, NJ 08854, USA; 5 Northport VA Medical Center, Northport, NY 11768, USA

**Keywords:** PIK3CA, STK11, lung cancer, LUAD, KRAS, orthotopic model

## Abstract

Constitutively active KRAS mutations are highly prevalent in lung cancers, but the direct role of its downstream phosphatidylinositol 3-kinase (PI3K) pathway in tumor progression remains unclear. A previous study established the requirement for PIK3CA, the alpha catalytic isoform, in lung tumor development in mouse models with an intact *Trp53* tumor suppressor. In this study, we further investigated the requirement of PIK3CA for tumor growth both *in vitro* and *in vivo*. We first generated a “KPA” cell line by genetically deleting *Pik3ca* from a murine lung adenocarcinoma “KP” cell line harboring oncogenic *Kras^G12D^* and lacking *Trp53*. We also examined the requirement for STK11, a tumor suppressor and metabolic regulator frequently co-mutated with KRAS in lung cancer. We found that *Pik3ca* is not required for cell survival and growth *in vitro*, even under anchorage-independent conditions, but reduced the growth rate by 15%. We next orthotopically implanted KP and KPA cells into syngeneic mice and found that PIK3CA is absolutely required for tumor progression, even in the absence of *Trp53*. Implantation of KP cells, or a “KPS” cell line lacking the *Stk11* gene, led to rapid tumor growth and death of all host animals. In contrast, mice implanted with KPA cells all survived with no detectable lung tumors. The gene expression profiles from cultured cell lines suggest oxidative stress as a potential vulnerability of KPA cells. Indeed, we found KPA cells were more sensitive to hydrogen peroxide and diethyl maleate-induced oxidative stress as compared to KP and KPS cells. Together, these results indicate that PIK3CA is not required for lung cancer cell growth induced by mutant KRAS *in vitro* but is essential for *in vivo* progression and growth.

## 1. Introduction

Lung adenocarcinoma (LUAD) accounts for about 40% of non-small cell lung cancer (NSCLC) cases and often carries oncogenic KRAS mutations, including KRAS^G12C^, KRAS^G12D^, and KRAS^G12V^ [1,2]. While the canonical RAS pathway primarily activates the MAPK signaling cascade, the phosphatidylinositol 3-kinase (PI3K) pathway is also activated downstream of RAS signaling [3,4,5,6]. This occurs upon active GTP-bound KRAS binding and signaling through direct binding to the catalytic p110 subunit of the PI3K complex at the RAS-binding domain (RBD) [7]. In solid tumors, the predominant catalytic isoform of the PI3K pathway is the alpha isoform encoded by the PIK3CA gene, which is required for tumor growth and maintenance [5,6,8]. Activated PIK3CA produces phosphatidylinositol-3,4,5 triphosphate (PIP_3_) to activate AKT protein kinases and promote tumorigenesis and growth in many neoplastic diseases, including lung cancers [5,6,7]. We have previously demonstrated that *Pik3ca* ablation from pancreatic tumor cells results in the clearance of tumor cells by tumor-infiltrating T cells [3] and similarly, genetic deletion of the downstream Akt isoforms dramatically slows tumor growth in murine pancreatic adenocarcinoma [4], further exemplifying the importance of this pathway in cancer biology.

PI3K has been demonstrated to be essential for tumorigenesis and maintenance in a mouse *Kras^G12D^* LUAD model. More specifically, the induced disruption of the binding domain between KRAS and PIK3CA results in an inability for tumors to grow and causes regression of existing lung tumors [6,7,9]. The PI3K/AKT pathway has been extensively studied as a potential therapeutic target in lung cancer with limited success, partly due to systemic adverse effects of PI3K inhibition [8,10]. Inhibition of the downstream effectors, AKT and mTOR, has been increasingly investigated in recent years as monotherapy or in combination with PI3K inhibitors, with promising preclinical data [11]. However, clinical trial outcomes in NSCLC patients have been disappointing [12,13,14], although these trials have not prioritized the selection of biomarkers, such as specific KRAS mutations, which may improve efficacy in specific patient sub-populations. Therefore, more work needs to be done to understand the mechanistic role of PIK3CA in tumor progression downstream from KRAS binding to define the most appropriate treatment strategies.

Despite the importance of the PI3K/AKT pathway in LUAD, activating mutations in PIK3CA are uncommon, especially in KRAS-mutant LUAD [8,15,16]. In contrast, the loss of serine threonine kinase 11 (STK11), also known as liver kinase B1 (LKB1), is frequently found together with oncogenic KRAS mutations in LUAD [17] and typically confers a worse prognosis [1,18]. STK11 is well known to regulate cellular metabolism by activating AMPK in response to hypoxia, nutrient depletion, or increased oxidative stress [18]. AMPK, in turn, inhibits mTOR, another downstream effector of the PI3K/AKT pathway, which regulates cell metabolism and growth [18,19,20].

Since oncogenic PIK3CA mutations have been determined to similarly shift cells from oxidative to glycolytic metabolism in cancer [21,22], we were curious to see the effects of the loss of tumor *Pik3ca* or *Stk11* on lung tumor progression. To study this, we constructed and characterized a *Kras^G12D^ Trp53^−/−^* mouse LUAD orthotopic implantation model in an immunologically intact host mouse with loss of tumor *Pik3ca* or *Stk11* to explore the effects of each gene independently in tumor growth and progression.

## 2. Materials and Methods

### 2.1. Cell Lines—Source and Generation

#### 2.1.1. Generation of KP Cells

The parental mouse lung cancer cell line was derived from tumor-bearing lungs of a *Kras^LSL-G12D/+/^Trp53^flox/flox^* mouse in C57BL/6J (B6) (Jackson Laboratory, Bar Harbor, ME, USA) that was infected intranasally with adenovirus expressing Cre recombinase, resulting in the expression of one *Kras^G12D^* allele and ablation of both *Trp53* gene copies in lung cells [23,24]. The cells were then infected with a lentivirus expressing firefly luciferase (Cellomics Technology PLV-10064, Halethorpe, MD, USA), and clones were selected using G418. The clone with the highest luciferase expression, which we refer to as KP, was selected for subsequent experiments. Tumorigenicity of the KP cells was confirmed by injecting the cells through the chest wall into either lung of syngeneic B6 mice (Supplemental Figure S1).

#### 2.1.2. Generation of KPA Cells

KP cells were transfected with CRISPR/Cas9 knockout and Homology-Directed Repair (HDR) plasmids (Santa Cruz Biotechnology sc-422231-KO-2 and sc-422231-HDR-2, Dallas, TX, USA) to delete the *Pik3ca* gene. Clones were selected in puromycin and loss of PIK3CA expression was verified by western blotting.

#### 2.1.3. Subcloning of KPA Cells

Cells were reinfected with a lentivirus expressing firefly luciferase (Kerafast FCT230, Boston, MA, USA) and clones were selected using blasticidin. All experiments were performed using these selected subclones unless noted otherwise.

#### 2.1.4. Generation of KPS Cells

KP cells were similarly transfected with CRISPR/Cas9 knockout and HDR plasmids (Santa Cruz Biotechnology sc-423192 and sc-423192-HDR) to delete the *Stk11* gene. Transfected cells were selected in puromycin and loss of STK11 expression was verified by western blotting.

#### 2.1.5. Cell Culture

All cell lines were cultured in RPMI 1640 medium (Corning 10-040-CV, Tewksbury, MA, USA) containing 1% penicillin/streptomycin (Gibco 15140-122, Grand Island, NY, USA) and 10% fetal calf serum (Corning 35-015-CV) at 37 °C in a humidified atmosphere with 5% CO_2_. Cells were kept in culture for no longer than one month.

### 2.2. In Vitro Studies

#### 2.2.1. Cell Proliferation

For assessment of cell growth rates, cells were seeded in cell culture dishes (Corning 353001) at an initial density of 1.6 × 10^5^ (day 0) then counted at 24 hour intervals over 4 days. Growth medium was aspirated and replaced after 48 hours to maintain optimal growth conditions. At each time point, cells were trypsinized (Corning 25-053-CI), an aliquot mixed with an equal volume of trypan blue (Invitrogen T10282, Waltham, MA, USA) and at least 400 cells were counted with a hemacytometer. Growth curves were fit using a linear fit of the log-transformed cell counts for each day and plotted using GraphPad Prism (version 10.5.0, GraphPad, Boston, MA, USA).

#### 2.2.2. Western Blotting

Cells were rinsed twice with cold PBS (Corning 21-031-CV) and then lysed on ice with RIPA buffer containing 50 mM HEPES pH 7.5, 10 mM sodium pyrophosphate, 50 mM NaCl, 50 mM NaF, 5 mM EDTA, 1 mM sodium orthovanadate, 0.25% sodium deoxycholate, 1% NP-40, 1 mM phenylmethylsulfonyl fluoride, and 1 μL/mL protease inhibitor cocktail (Sigma-Aldrich P8340, St. Louis, MO, USA). Cell lysates were then cleared by centrifugation at 14,000× *g* for 20 minutes at 4 °C. The protein content of the resultant supernatant was measured using a Bradford assay (Bio-Rad, Hercules, CA, USA) on a SpectraMax M5 instrument (Molecular Devices, San Jose, CA, USA). Equal amounts of protein were combined with SDS sample buffer and separated by SDS-PAGE followed by semi-dry transfer onto nitrocellulose membranes. After blocking in 5% non-fat dry milk, membranes were incubated overnight at 4 °C with primary antibody in Tris-buffered saline plus 0.1% Tween 20. The blot was incubated for 1 h with Horseradish Peroxidase (HRP)-linked secondary antibody (GE Healthcare, Chicago, IL, USA) and signals were captured by a FluorChem M imager equipped with AlphaView 3.4.0.0 software (ProteinSimple, San Jose, CA, USA) using Western Lightning Plus ECL reagents (PerkinElmer NEL105001EA, Shelton, CT, USA) or SuperSignal West Femto (Thermo Scientific 34095, Waltham, MA, USA). To detect multiple proteins on one membrane, blots were cut into pieces that were probed with separate antibodies.

Antibodies to AKT1 (#2938), PIK3CA (#4249), phospho-Ser473 AKT (#4060), AMPK (#2532), and phospho-Thr172 AMPK (#2531) were purchased from Cell Signaling Technology (Danvers, MA, USA). Antibodies to STK11/LKB1 (sc-32245) and HSP90 (sc-13119) were purchased from Santa Cruz Biotechnology.

#### 2.2.3. 3D Cell Culture

Cells were 3D cultured as described by our lab previously [3,4]. Briefly, 1 × 10^3^ cells were suspended in culture medium with a methylcellulose solution (Sigma-Aldrich M0512-100G) with a final concentration of 0.24%. The cells were cultured by hanging drop method on the lid of a plate filled with PBS to avoid evaporation. The plate was incubated at 37 °C with 5% carbon dioxide for seven days and imaged at 40× magnification with light microscopy.

#### 2.2.4. MTT Assays

Cells were plated at a density of 5 × 10^3^ cells per well in a 96-well tissue culture-treated flat-bottom plate (Sarstedt 83.3924, Nümbrecht, Germany) and incubated for 48 hours. The cells were then treated with varying concentrations of hydrogen peroxide (Fisher Chemical H325-500, Waltham, MA, USA), diethyl maleate (DEM) (Fisher AC114440050), cisplatin (Adooq Bioscience A10221, Irvine, CA, USA), or docetaxel (MedChemExpress HY-B0011, Monmouth Junction, NJ, USA) for 24 or 48 hours. Cells were also pre-treated for 24 hours with 400 nM alpelisib (MedChemExpress HY-15244) or dimethyl sulfoxide (DMSO) vehicle then the media was replaced with 250 μM DEM or 100 μM hydrogen peroxide for an additional 24 hours. All drugs were solvated in 100% DMSO with final assay concentrations being less than 0.01% DMSO. 3-[4,5-dimethylthiazol-2-yl]-2,5-diphenyl tetrazolium bromide (MTT)-based assays were then performed as previously described [25]. After addition of a 5 mg/mL MTT solution (Alfa Aesar L11939.03, Ward Hill, MA, USA), the plates were incubated for 1 hour at 37 °C, 5% CO_2_. Formed insoluble formazan crystals were dissolved in DMSO (Thermo Scientific 036480.AP) and the absorbance was read at 570 nm with a 690 nm background subtraction on a Spectromax M5 (Molecular Devices).

#### 2.2.5. Statistics

All statistics were calculated using GraphPad Prism using a one- or two-way ANOVA with a Bonferroni post hoc test and a significance value of 0.05. Survival curves were analyzed in the same software with a log-rank test.

### 2.3. In Vivo Studies

#### 2.3.1. Orthotopic Implantation of Tumor Cells in the Lung

All procedures involving live animals were performed according to institutional policies at Northport VAMC (ACORP) and Stony Brook University (IACUC).

Cells to be used for orthotopic implantation were trypsinized and extensively washed with cold PBS by repeated centrifugations at 300× *g* for 5 minutes at 4 °C. The number of viable cells was then counted in the presence of Trypan Blue using a Countess II Automated Cell Counter (Invitrogen). The injection solution was prepared by suspending 1 × 10^7^ viable cells per mL in PBS containing 0.5 mg/mL of Matrigel (Corning 356231), yielding 5 × 10^5^ cells in a 50 µL injection, and kept on ice until implantation. All steps were carried out under aseptic conditions.

Cells were implanted in the lung of 8- to 14-week-old male C57BL/6J (B6) mice or SCID (B6.CB17-Prkdc^scid^/SzJ) mice purchased from Jackson Laboratories. Mice were anesthetized with 2–4% isoflurane. A 5 mm incision was made on the skin over the left lung, and the underlying muscles were separated to visualize the ribs and motion of the left lung unless otherwise specified. A 28-gauge needle attached to a ½ cc U-100 insulin syringe was inserted to a depth of about 3 mm at the mid-axillary line and 50 μL of injection solution was injected into the upper left lung, a volume selected to maintain appropriate cell density while remaining within reported tolerable pulmonary injection volumes in mice [26,27]. The wound was closed with a 9 mm wound clip. Post-procedure, mice were injected subcutaneously with 2 mg/kg ketorolac for pain relief and 0.1 mL of 2.27 mg/mL enrofloxacin antibiotic. After implantation, the animals were observed for 30–60 minutes to monitor their recovery.

#### 2.3.2. Animal Survival Studies

After implantation, mice were monitored for signs of distress and weighed at least weekly or more frequently if persistent weight loss was noted. Mice were euthanized if they lost 15% or more of their initial weight and/or appeared to be in severe distress as determined by a blinded observer. A necropsy was performed to confirm the presence of gross tumors. For some experiments, tumors or lungs were subsequently dissected, fixed for 24 hours in a 10% buffered formalin solution, and then embedded in paraffin blocks. Kaplan–Meier survival curves were generated and plotted using GraphPad Prism.

#### 2.3.3. *In Vivo* Luminescence Imaging (IVIS)

For the initial tumor growth and progression study of the parental KP cells, tumor progression was monitored using a Lumina III In Vivo Imaging System (IVIS, PerkinElmer) as previously described by us [3].

#### 2.3.4. H&E Staining

H&E staining of suspected tumors fixed and embedded in paraffin blocks was performed at HistoWiz, Inc. (Long Island City, NY, USA), using the Leica Bond RX automated stainer (Leica Microsystems, Wetzlar, Germany) and a standard protocol. Blocks were sectioned at 4 μm then slides were dewaxed using xylene and alcohol-based dewaxing solutions. Tissue was incubated with hematoxylin according to the manufacturer’s protocol. The slides were visualized using an Aperio GT 450 DX slide scanner (Leica Biosystems) at 40×. The resultant images were analyzed for identification of tumor sections.

### 2.4. Bulk-RNA Sequencing

Cells were cultured to 80–90% confluence in 10 cm cell culture dishes (Sarstedt 83.3902), washed with cold PBS, and lysed using 600 μL RLT Plus buffer (Qiagen 1030963, Venlo, The Netherlands) +1% *v*/*v* β-mercaptoethanol (Sigma-Aldrich M6250-100ML) and the lysate homogenized using QIAshredder (Qiagen 79654), following the manufacturer’s instructions. Total RNA was extracted using the Qiagen AllPrep DNA/RNA/miRNA Universal Kit (Qiagen 80224) and quantified using a Nanodrop 2000 (ThermoFisher, Waltham, MA, USA).

RNA integrity was analyzed on a TapeStation 4200 (Agilent, Santa Clara, CA, USA), and the RNA Integrity Numbers (RINs) were 9.8 and higher for all samples used. Bulk RNA library preparation and sequencing was performed by a commercial supplier (Novogene, Inc., Sacramento, CA, USA), using the NEBNext^®^ Ultra™ II RNA Library Prep Kit for Illumina (New England Biolabs E7770, Ipswich, MA, USA) with the NEBNext^®^ Poly(A) mRNA Magnetic Isolation Module (New England Biolabs E7490). Libraries were sequenced at a depth of approximately 33 million paired reads per sample (PE150) on an Illumina NovaSeq X Plus Instrument (Illumina, San Diego, CA, USA).

#### RNA-Seq Data Analysis

The transcripts were quantified and mapped to the GRCm39 (GCA_000001635.9) reference genome from ENSEMBL using Salmon with correction for GC bias. The resultant quantification files were loaded into R (version 4.5.1) in RStudio (version 2024.12.1, build 563), and the DESeq2 package (version 1.48.1) [28] identified differentially expressed genes. To produce a heatmap with the most differentially expressed genes between cell types with the least variation between replicates with the geneFilter package (version 1.90.0), the data was transformed with a variance-stabilizing transformation, but all further analyses were done with the untransformed DESeq2 results to limit bias. The results were filtered to only include genes with a *p*-value less than 0.01, an absolute log_2_-fold change greater than one, and a count of at least 250. This filtered data was used with the gseGO function of clusterProfiler (version 4.16.0) [29] and fgsea (version 1.34.2) to find the gene ontology enriched by molecular function with a more relaxed *p*-value limit less than 0.05.

## 3. Results

### 3.1. Loss of Pik3ca, but Not Stk11, Moderately Reduces In Vitro Growth Rate in Murine Kras-Mutant Lung Adenocarcinoma Cell Line

**Figure 1:**
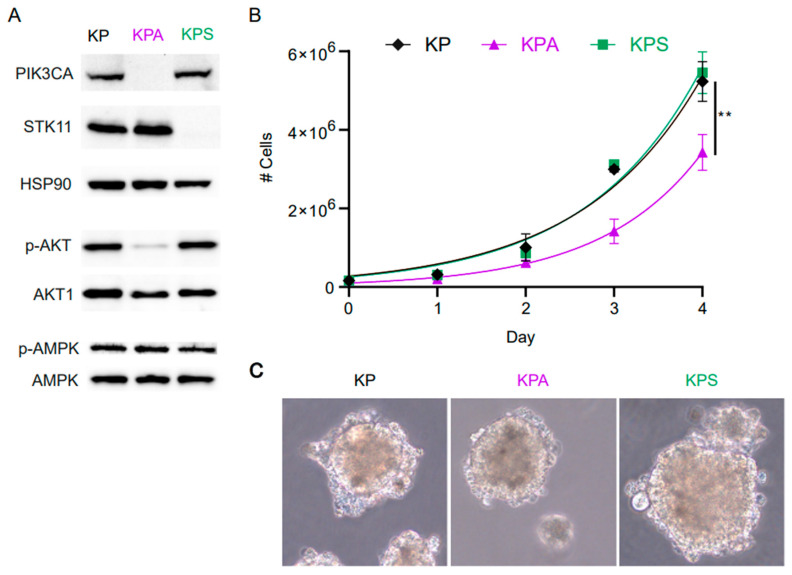
Generation and in vitro characterization of KP, KPA, and KPS cell lines. (**A**) Western blot of parental KP, derived KPA (*Pik3ca^−/−^)*, and KPS (*Stk11^−/−^*) cells with HSP90 as loading control. (**B**) Proliferation of KP, KPA, and KPS cells *in vitro*. Shown are averages of 3 separate experiments with the standard error of the mean. Growth at each day compared by two-way ANOVA with Bonferroni post hoc for day 4 (** *p* < 0.001 KP vs. KPA). (**C**) Representative light-microscopy images of spheroid growth of KP, KPA, and KPS cell lines after 7 days of growth in 3D-methylcellulose culture (40× magnification).

To study the effect of loss of PIK3CA in mutant KRAS lung cancer, we developed a murine tumor cell line (KPA) lacking this protein from a parental cell line (KP), which we obtained through isolation of lung tumor cells from a *Kras^LSL-G12D/+^/Trp53^flox/flox^* B6 mouse after intranasal infection with Cre-expressing adenovirus and subsequent transfection with a luciferase gene to non-invasively track longitudinal tumor progression. A CRISPR-Cas9 system was then used to genetically delete *Pik3ca* from KP cells to create the “KPA” cell line. We also used the same CRISPR-Cas9 technique to produce a *Stk11*-deficient “KPS” cell line from KP cells to study the effects of loss of STK11 in mutant KRAS lung cancer. We validated PIK3CA and STK11 deletion by western blotting (Figure 1A). Additionally, deletion of the target genes resulted in decreased phosphorylation of AKT in KPA or AMPK in KPS cell lines, respectively (Figure 1A), consistent with canonical signal transduction from PI3K to AKT [3,8,9,30] and STK11 as one of the kinases that phosphorylates AMPK [18].

We first measured the *in vitro* proliferation rates over several days and compared them to the growth of the parental cell line, KP (Figure 1B). While the KP and KPS cells maintained a nearly identical doubling time of 0.75 and 0.74 days, respectively (95% confidence interval (CI) (0.69–0.83 and 0.67–0.80), the growth rate of the KPA cells diverged at day 2 with an overall 15% slower doubling time of 0.86 days (95% CI 0.77–0.97) at day 4 when compared to the KP and KPS cell lines (Figure 1B). Next, we tested the ability of each cell line for anchorage-independent growth by growing the three cell lines in a methylcellulose-based 3D culture, as previously described [3]. Over the course of a week, all the cell lines grew into spheroids, indicating that while the cells are adherent in culture conditions, anchorage is not a requirement for cell growth in any of the cell lines (Figure 1C).

### 3.2. Loss of PIK3CA but Not STK11 Markedly Decreases Tumor Growth and Progression In Vivo

To investigate the distinct roles of *Pik3ca* and *Stk11* in a physiologically relevant model of LUAD, we implanted the three cell lines orthotopically into the lungs of mice. Identical numbers of KP, KPA, or KPS cells were implanted into syngeneic B6 mice and monitored without further treatment until death or measurable, pre-specified end points, including excessive weight loss or signs of distress.

**Figure 2:**
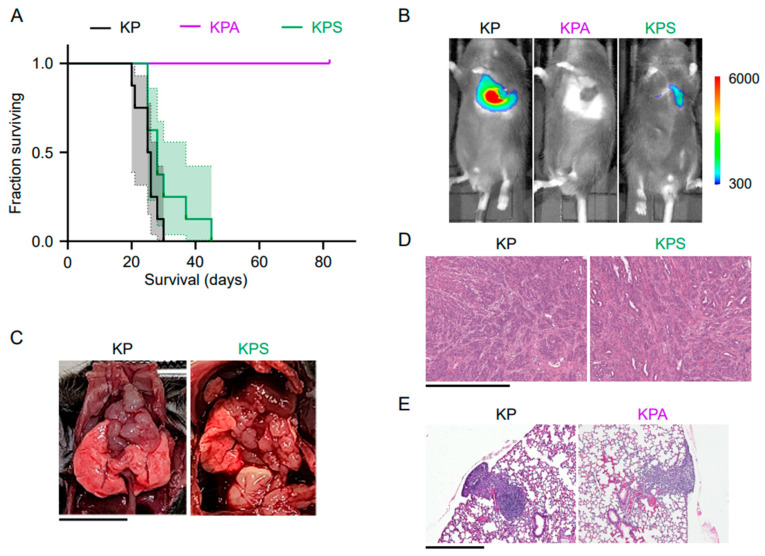
*In vivo* tumor progression of implanted KP, KPA, and KPS cells in murine lung adenocarcinoma model. 5 × 10^5^ KP, KPA, or KPS cells were implanted into the lungs of mice in a representative experiment. (**A**) Kaplan–Meier survival curves for mice implanted with KP (*n* = 8), KPA (*n* = 7), or KPS (*n* = 8). Median survival: KP, 25.5 days; KPS, 28 days; all KPA alive on day 82. *p* < 0.0001, KP versus KPA (log-rank test). Shaded regions represent 95% confidence interval for each implanted cell line. (**B**) Representative tumor progression observed with IVIS imaging 18 days post-implantation. Scale represents luminescence intensity (arbitrary units). (**C**) Representative post-mortem analysis of sacrificed KP- and KPS-implanted mice. Scale bar represents 10 mm. (**D**) Representative H&E staining confirming tumors in KP and KPS-implanted mice. Scale bar represents 500 μm. (**E**) To assess early tumor development, 5 × 10^5^ KP or KPA cells of earlier subclones were implanted into the lungs of syngeneic mice (*n* = 3 mice per group). At 10 days post-implantation, the lungs were fixed and sectioned. Shown are representative H&E stained sections of lungs from each group of mice. Scale bar represents 500 μm.

KP- and KPS-injected mice had similar overall survival (Figure 2A) with KP-injected mice surviving a median of 25.5 days (95% CI 20–28 days) and KPS-injected mice surviving a median of 28 days (95% CI 25–37 days). In contrast, none of the KPA-injected mice died or reached pre-specified endpoints before the end of the study (82 days post-implantation) (Figure 2A). These median survival times are consistent with other independent experiments with paired implantation of KP and KPS or KP and KPA cells (data available upon request).

*In vivo* fluorescence imaging was used to detect the luciferase signal from implanted cells for non-invasive, weekly tumor monitoring post-implantation with In Vivo Imaging System (IVIS) imaging (Figure 2B). At 18 days post-implantation, KP- and KPS-injected mice typically exhibit obvious tumor development localized around the lungs, albeit with a stronger luminescent signal in mice injected with KP cells than KPS cells (Figure 2B). Sacrificed mice that exhibit a strong luminescent signal on IVIS reveal extensive development of large masses around the lungs, mediastinum, and diaphragm (Figure 2C) which can be confirmed through microscopic examination to be tumors with similar morphology between KP- and KPS-implanted mice (Figure 2D). Conversely, KPA-injected mice show no clear evidence of macroscopic tumor growth through the end of the study (Figure 2B).

Since KPA cells were previously demonstrated to have the ability to grow anchorage-independently in culture, we looked more specifically at the early lung tumor development of KPA cells to confirm that the tumors could engraft in the lung. To do this, we harvested the lungs of mice orthotopically implanted with KP or KPA cells at 10 days post-implantation (Figure 2E). None of the KP- or KPA-injected mice had visible tumors on the surface of the lungs, but microscopic evaluation of the H&E-stained sections revealed clear tumor growth in all mice implanted with KP cells and in 2 out of 3 mice implanted with KPA cells (Figure 2E).

This result resembles our previous findings, where PIK3CA ablation resulted in T-cell-mediated clearance of pancreatic tumors and, therefore, improved survival [3]. To investigate if a similar immune mechanism is occurring in this lung cancer model, we implanted KPA cells into SCID mice in the B6 genetic background that lack a functional adaptive immune system. SCID mice implanted with KPA cells also did not form tumors, and all host animals survived until the end of the study, like the KPA-implanted B6 mice (Supplemental Figure S2A). To rule out that these results were due to a requirement for a higher starting number of implanted KPA cells, we repeated the experiment with a 10-fold higher cell count and found the equivalent 100% host animal survival (Supplemental Figure S2B). This indicates that, although KPA tumors can engraft in the lungs of mice (Figure 2E), they fail to progress by some mechanism distinct from adaptive immune clearance. In summary, these results align with the previously established requirement of PIK3CA binding to KRAS for in vivo tumor progression [6,7] but raise additional questions on the mechanism for the KPA cells’ ability to grow *in vitro* but not progress *in vivo*.

### 3.3. Cells Deficient in Pik3ca Have Reduced Gene Expression in Key Oxidative Stress Pathways

To identify potential mechanisms underlying the lack of *in vivo* lung tumor progression of KPA tumor cells, we compared the gene expression profiles of cultured KP, KPA, and KPS cells using bulk RNA sequencing. We transformed the data with a variance stabilizing transformation to identify the most differentially expressed genes (DEGs) between cell types with the least variation between replicates to yield the most robust, consistent changes. Next, we performed gene set enrichment analysis (GSEA) to determine if there were any other molecular changes that were significantly different between the cell lines. When all DEGs were included in this analysis, the most changed pathways in either KPA or KPS in comparison to KP were identical (data available upon request), so we stringently filtered the gene list to prioritize DEGs with a large, highly significant log_2_-fold change compared to KP cells to ensure we only included robust and specifically different genes in GSEA.

**Figure 3:**
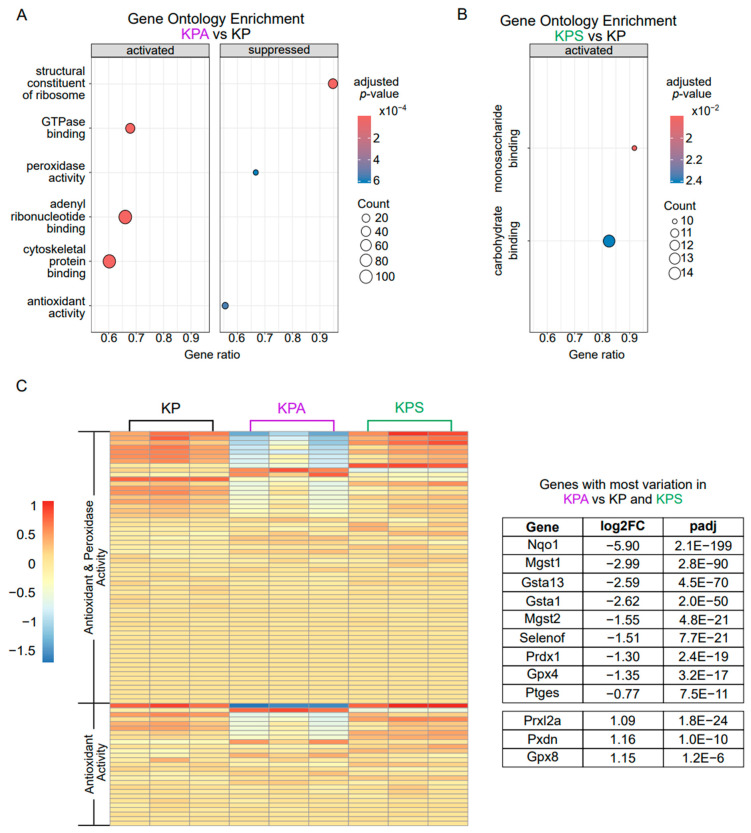
RNA sequencing comparison of pathway enrichment. (**A**,**B**) RNA was harvested from cultured cell lines and sequenced. Gene set enrichment analysis (GSEA) of the top activated and/or suppressed molecular functions by gene ontology in KPA (**A**) and KPS (**B**) compared to KP. (**C**) Heatmap of variance-stabilized differential expression of all genes in the antioxidant activity (GO:0016209) and peroxidase activity (GO:0004601) pathways (left). The genes with the greatest variability in expression in the KPA cell line in comparison to both other cell lines are presented in the table on the right with the log_2_ fold change (log2FC) and adjusted *p*-value (padj) in comparison to the KP cell line.

From the remaining list of 1830 and 592 DEGs in KPA or KPS cells, respectively, we then analyzed the enriched molecular function gene ontology terms to identify differences between molecular processes which may contribute to the disruption of *in vivo* tumor growth. KPA cells had decreased activity in both peroxidase and antioxidant pathways (Figure 3A) while KPS cells only had two significantly activated functions of monosaccharide and carbohydrate binding (Figure 3B). This suggests that loss of PIK3CA, but not STK11, from LUAD tumor cells may affect the redox balance and/or the metabolic response to oxidative stress.

To gain more insight into how KPA cells’ peroxidase and antioxidant gene expression differed from the KP and KPS cell lines, we compared the expression of all genes within these pathways. Peroxidase activity is a subset of antioxidant activity, so only overlapping genes specific to the antioxidant activity pathway are presented. Approximately half of the genes had any significant variation between the three cell lines and, in accordance with the overall downregulation of both pathways seen in Figure 3A, most of these genes are downregulated in KPA cells compared to KP cells (Figure 3C). For the genes with the greatest variation between all three cell lines, KPA cells had a consistently opposite direction of gene expression changes compared to both KP and KPS cells. This suggests KPA cells may have a reduced ability to manage reactive oxygen species (ROS) and maintain redox balance, which may contribute to the inability of these cells to generate tumors *in vivo* (Figure 3C). This is supported by the specific decrease in gene expression for several downstream targets of the transcription factor NRF2, a redox-responsive regulator of antioxidant and detoxification responses, including glutathione transferases (*Mgst1*, *Gsta1*, *Mgst2*, and *Gsta13*), a lipid hydroperoxide-reducing glutathione peroxidase (*Gpx4*), and oxidoreductases (*Nqo1* and *Prdx1*) [31,32] (Figure 3C).

### 3.4. Inhibition of PIK3CA Activity Increases Sensitivity to Oxidative Stress but Enhances Resistance to Cytotoxic Chemotherapies

ROS have a well-established involvement in KRAS-driven lung cancers, and oncogenic Ras, including KRAS, is well known to promote constitutive production of ROS [33]. In addition, the lung parenchyma has a higher oxygen concentration than any other organ and thus contains additional regulatory mechanisms for oxygen sensitivity [34,35]. Therefore, lungs are proposed to have specialized responses to ROS in comparison to other organs, although the details of this are incompletely understood [35,36].

Overactivation of the PI3K/AKT pathway requires upregulated ROS detoxification to support tumor progression through stabilization and activation of NRF2 [22]. Although loss of STK11 is associated with metabolic reprogramming in NSCLC, this is through reduced lipid metabolism rather than increasing anaerobic glycolysis [37]. Therefore, we hypothesized that the lack of KPA tumor progression *in vivo* may result from an increased vulnerability to oxidative stress due to an impaired capacity for ROS detoxification.

**Figure 4:**
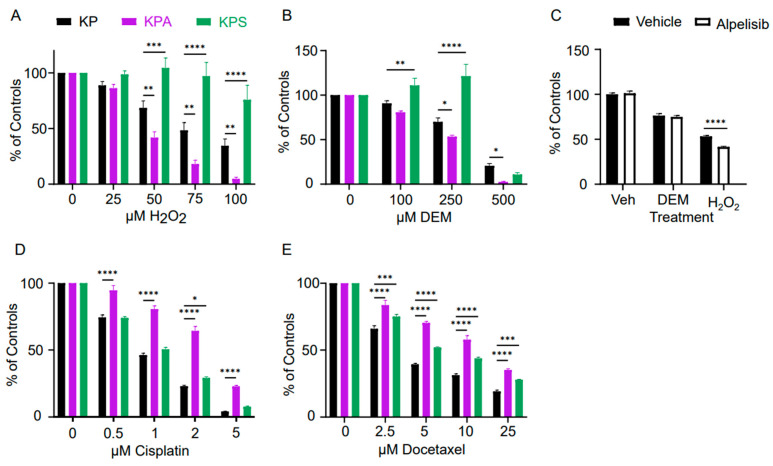
Effects of hydrogen peroxide, chemotherapy, and glutathione depletion on KP, KPA, and KPS cells. (**A**,**B**) Cell lines were treated with increasing doses of hydrogen peroxide (**A**) or diethyl maleate (DEM) (**B**) for 24 h and the metabolic activity of the cells was measured by MTT assay. Plotted is the mean and standard error of absorbance at 570 nm from five replicates across 4 independent experiments. (**C**) KP cells were pre-treated with 400 nM alpelisib or vehicle for 24 h then treated with 250 µM DEM, 100 µM hydrogen peroxide, or vehicle for an additional 24 h and the metabolic activity measured with MTT assay. Plotted is the mean and standard error of absorbance at 570 nm from twenty replicates. (**D**,**E**) Cell lines were treated with a dose–response of cisplatin (**D**) or docetaxel (**E**) for 48 h and the metabolic activity assessed by MTT assay. Plotted is the mean and standard error of absorbance at 570 nm from 5 replicates across 4 independent experiments. In all experiments, the variance at each dose was compared against KP with a two-way ANOVA and Bonferroni post hoc * *p* < 0.05; ** *p* < 0.01, *** *p* < 0.005, **** *p* < 0.0001.

To test the sensitivity of each cell line to ROS, we treated KP, KPA, and KPS cells *in vitro* with hydrogen peroxide (H_2_O_2_). Extracellular treatment with H_2_O_2_ increases intracellular ROS levels and, at high doses, induces cell death [38]. Therefore, we exposed KP, KPA, and KPS cells to varying concentrations of H_2_O_2_ and measured the residual metabolic activity using MTT assays. While KPA cells were markedly more sensitive to H_2_O_2_ than KP cells, KPS cells were dramatically less sensitive (Figure 4A). For KP cells, loss of approximately 50% metabolic activity (IC_50_) compared to control-treated KP cells occurred around 82.5 μM of H_2_O_2_ (95% CI: 66.1 to 104). In KPA cells, the IC_50_ was approximately 35.1 μM of H_2_O_2_ (95% CI: 28.7 to 42.7), meaning that KPA cells are about 43% more sensitive to ROS than KP cells. At the highest tested concentration of 100 μM H_2_O_2_, KPS cells only decreased to 76% metabolic activity with respect to control-treated KPS cells, making the IC_50_ indeterminable from this data (Figure 4A). This aligns with previous studies demonstrating that STK11-deficient mouse embryonic fibroblasts increase intracellular ROS accumulation levels by two-fold compared to wild-type cells in response to treatment with a sub-cytotoxic dose of H_2_O_2_ [39].

Glutathione acts as a reducing agent to detoxify ROS and maintain redox balance within the cellular environment [40]. Diethyl maleate (DEM) is a commercially available electrophile that alkylates reduced intracellular glutathione to block its antioxidant activity [38,41,42]. Therefore, treatment with DEM can inhibit the cellular antioxidant response, and hence, potentially increase sensitivity to ROS. Our results revealed that both KP and KPA cells had a dose-dependent response to DEM treatment with the IC_50_ for KP being 353 μM (95% CI: 290 to 423) and KPA being significantly more sensitive with an IC_50_ of 201 μM (95% CI: 169 to 237) (Figure 4B). While the KPS cells also showed some dose-dependent response to DEM, the behavior was very different with metabolic activity slightly increasing with DEM treatments up to a concentration of 250 μM and then plummeted to 10.7% metabolic activity of the control with the 500 μM dose, possibly due to a shift from oxidative to glycolytic metabolism to account for decreased mitochondrial spare respiratory capacity [43] (Figure 4B).

To determine if the lack of PI3K catalytic activity is sufficient to explain the altered response to ROS in KPA cells, we pharmacologically inhibited PIK3CA activity in KP cells with alpelisib. This drug, commercially known as Piqray^®^, is a potent selective PIK3CA inhibitor, which blocks the kinase activity of PIK3CA and is currently used in the treatment of breast cancer [44,45]. We treated KP cells with DEM and H_2_O_2_, with and without alpelisib, to test whether the inhibition of PIK3CA kinase activity would be sufficient to promote ROS sensitivity of KP cells—either directly or via glutathione depletion—to the levels seen in KPA cells. At a dose of 250 μM DEM, there is no difference in the metabolic activity levels of KP cells with or without alpelisib treatment (Figure 4C). By contrast, alpelisib treatment caused the KP cells to be significantly more sensitive to 100 μM H_2_O_2_ than vehicle-treated control cells (Figure 4C). The combined treatment of alpelisib and H_2_O_2_ only resulted in a 58% loss in metabolic activity (Figure 4C), whereas treatment of KPA cells with the same concentration of H_2_O_2_ yielded a 95% loss (Figure 4A). Taken together, these findings suggest that pharmacological inhibition of PI3K kinase activity alone is insufficient to completely describe the effect of the loss of PIK3CA expression on ROS sensitivity.

We next tested whether the increased sensitivity to drug treatments in KPA cells was a generalizable, nonspecific drug effect by testing sensitivity to traditional cytotoxic chemotherapeutic agents. We treated KP, KPA, and KPS cells with different concentrations of cisplatin, a key component of cytotoxic chemotherapy for NSCLC [46,47,48,49]. Treatment of each cell line with cisplatin demonstrated strong resistance of KPA cells to the drug in comparison to KP and KPS cells, which demonstrated similar levels of sensitivity to each other (Figure 4D). The estimated concentration of cisplatin which reduced metabolic activity to 50% of control untreated KP cells (IC_50_) for KP and KPS cells overlapped with IC_50_ values of 0.85 (95% CI: 0.72 to 1.0) and 0.98 μM of cisplatin (95% CI: 0.86 to 1.1), respectively (Figure 4D). In contrast, KPA cells were significantly more resistant to cisplatin with an IC_50_ of 3.0 μM (95% CI: 2.4 to 3.7).

Similarly, we also exposed cells to docetaxel, a member of the taxane family and another commonly employed chemotherapy for NSCLC [50]. Treatment of KP, KPA, and KPS cells with docetaxel resulted in a dose-dependent decrease in metabolic activity across all cell lines (Figure 4E). For KP cells, the IC_50_ of docetaxel was 4.3 μM (95% CI: 3.8 to 4.7). KPS cells had a slightly higher IC_50_ of 7.1 μM (95% CI: 6.4 to 7.8), indicating slightly less sensitivity to docetaxel compared to KP at all tested concentrations. Like cisplatin, KPA cells showed significant resistance to docetaxel with an IC_50_ of 13 μM (95% CI: 12 to 15), over 300% less sensitive to docetaxel than KP cells (Figure 4E). In summary, KPA cells exhibited increased sensitivity to ROS stress but were more resistant to traditional cytotoxic chemotherapy compared to KP or KPS cells. This suggests that increased sensitivity to ROS from loss of PIK3CA activity is not a general increase in sensitivity to cellular stress but rather a specific response to ROS.

## 4. Discussion

The importance of the PI3K pathway in many cancers, including lung [8], pancreatic [3,51], colorectal [16], and cervical cancers [52], is well known. Previous efforts in understanding PIK3CA in KRAS-driven NSCLC have largely focused on the clinically relevant overactive PIK3CA phenotype from well-characterized mutations [8,15,16,45,53], but disruption of PIK3CA signaling in a mouse model has also revealed its necessity for tumor growth and maintenance [6,7,9]. More specifically, disruption of the RBD of PIK3CA abrogates tumor progression and reduces the size of existing lung tumors [6]. This suggests that pharmacological inhibition of PIK3CA might be a viable treatment option for KRAS-driven cancers. However, clinical attempts at pharmacologically inhibiting PI3K activity have been unsuccessful due to toxicity of systemic PI3K inhibition and possible putative compensatory activation of alternative cell signaling pathways [8,10,54], indicating the need for further investigation regarding the broader implications of reduced PIK3CA expression and/or activity. Therefore, we established and characterized a murine syngeneic orthotopic implantation *Kras*-mutant *Trp53*-deficient LUAD model to better understand the role of PIK3CA within the context of LUAD. To apply the model, we developed a KRAS mutant PIK3CA-null KPA tumor cell line. We also looked at STK11, whose loss is well established to result in more aggressive LUAD through shifting metabolic dependencies [1,18], with a KRAS mutant STK11-null KPS tumor cell line.

Previous methods to test the requirement for PI3K activity in lung cancer have involved mouse models with germline deletions or inducible modifications of PIK3CA [6,7,9]. These models were instrumental to the initial understanding of how KRAS and PIK3CA bind and interact to transduce signaling and affect *in vivo* tumor growth but have limited potential for further *in vivo* studies and are unable to examine the mechanisms precisely. Additionally, loss-of-function mutations in *TP53* are frequently seen in conjunction with KRAS mutation in human LUAD and result in 30% worsened overall survival [55]—a finding not mimicked in these models, which also limits the ability to generate cell lines from resultant tumors [56]. *In vitro* studies with primary embryonic fibroblasts from mice with mutated PIK3CA RBDs report accumulation of cells in the G1 and G2 phases of mitosis and reduced cell division in comparison to wild-type cells. The mutated fibroblasts lose the capacity for anchorage-independent growth [7], so our ability to generate and grow the KPA cell line was unexpected and indicates that PIK3CA is not a requirement for *in vitro* growth. Unlike KP and KPS cells with similar growth rates, we observed a small but significant decrease in the *in vitro* growth rate of KPA cells compared to KP cells. Furthermore, both KPA and KPS cells demonstrated anchorage-independent growth. This provides an interesting potential opportunity to study the role of PIK3CA in lung cancer, given that similar models of pancreatic [3] and lung cancer with silenced oncogenic KRAS [57] failed to grow *in vitro* or *in vivo*.

Therefore, we took advantage of the demonstrated *in vitro* growth ability and orthotopically implanted the KP, KPA, and KPS cells into the lungs of wild-type B6 mice with intact immune systems to investigate the growth rates in a more biologically relevant context. Although KP and KPS tumors grew at similar rates with similar survival curves, KPA tumors failed to progress *in vivo* resulting in a marked increase in survival in comparison to KP- and KPS-injected mice. The observed reduced *in vitro* growth rate alone is insufficient to account for the prolonged survival and lack of tumors. Additionally, KPA cells are able to successfully implant into the lungs yet are unable to progress even in the absence of a functional adaptive immune response in contrast to a similar murine model of pancreatic cancer [3,58]. Taken together, this indicates that PIK3CA, but not STK11, is required for growth and progression *in vivo* in KRAS-mutant NSCLC but dispensable for *in vitro* growth. This contrasts with activating KRAS mutations where our efforts to revert the oncogenic KRAS mutation present in KP cells back to wild-type were met with failure due to a lack of *in vitro* growth (data available upon request). Therefore, we began investigating the potential mechanisms driving the observed differences between the *in vivo* and *in vitro* behavior of KP, KPA, and KPS tumor cells with a focus on KPA cells.

By analyzing the transcriptomic profiles of the cultured cell lines, only the KPA cells presented reduced expression across peroxidase and antioxidant activity pathways, indicating a potential vulnerability to redox stress. Redox homeostasis is essential in healthy cells to maintain the balanced production of ROS, generated in the mitochondria as a byproduct of aerobic cellular respiration, with degradation by antioxidants to avoid excessive oxidative stress [38,41,59]. Cancer cells often have higher endogenous ROS levels than non-cancerous cells and use this as a survival mechanism to promote proliferation, but there exists a threshold of ROS at which the benefits are surpassed and cells will die [60]. Accordingly, ROS can have either pro- or anti-tumor effects depending on the local concentration and specific microenvironment of the tumor [61]. Induction of excessive ROS in cancer cells has been reported to reduce cell proliferation in a PI3K/AKT-dependent manner [18,60,62]. Therefore, accumulation of ROS to cytotoxic levels is a mechanism for killing aberrantly proliferative cells and a common indirect therapeutic strategy to halt tumor progression in oncology [59]. However, in many cancers, metabolic reprogramming is utilized by tumor cells to generate additional antioxidants and redox cofactors in the increasingly stressed, hypoxic microenvironment generated through malignant tumorigenesis [21,38,42,53,59,63,64]. Based on the genes that varied most from the KPA cell line in comparison to the other cell lines, there may be a deficiency in NRF2 sensing of oxidative stress and activation of antioxidant enzyme expression [31]. KRAS oncogenic mutations have been demonstrated to promote tumor development through the activation of antioxidant genes by NRF2 [65], making this a potential mechanism for the lack of KPA tumor progression *in vivo*. However, many of these genes have diverse functions in the cell, such as *Nqo1*, which acts as a superoxide reductase, but also associates with microtubules during mitotic spindle formation [66]. Additionally, some of the downregulated genes in KPA cells, particularly *Gpx4*, point toward ferroptosis or autophagy inhibition as other potential mechanisms [65,67]. More work is required to clearly identify the precise cellular process that differs in KPA cells from KP and KPS cells resulting in failure to progress *in vivo*.

We found that loss of PIK3CA strikingly increased sensitivity to ROS via hydrogen peroxide exposure, in contrast to loss of STK11 which exhibited marked resistance when compared to the parental KP cells. KRAS overactivation, which exists across all tested cell lines, is known to induce tumorigenicity with requirements for mitochondrial production of ROS and glutamine catabolism [68,69,70]. Similarly, the depletion of glutathione, an antioxidant produced from glutamine [22], with DEM resulted in the same inverse effects on metabolic viability between KPA and KPS cells. DEM increases ROS generation through glutathione depletion and inhibits the cell cycle [71]. While little is known about the specific effects of decreased expression of PIK3CA on metabolic changes in cancers, there is existing evidence that hyperactivated PIK3CA shifts glutamine metabolism in cancer [21,22,53]. Across multiple human cancer cell lines, hyperactivated PIK3CA in tumors requires 2-oxoglutarate dehydrogenase for survival and proliferation due to increased glucose metabolism and a reduced NAD+/NADH ratio [53]. Accordingly, glutathione biosynthesis, simulated by oncogenic PI3K/AKT signaling, has been shown to trigger metabolic reprogramming in breast cancer [22]. By contrast, STK11 has a well-documented role in metabolic reprogramming [18,43,63,64]. STK11 regulation of AMPK is essential for maintaining nicotinamide adenine diphosphate (NADPH) homeostasis and increasing glucose catabolism [64], whereas loss of STK11 increases HIF-1α with increased mTOR expression and higher ROS, which results in a more glycolytic-dependent metabolism [63]. In support of our findings, KRAS mutation with STK11 deletion has been reported to maximize metabolic capacity, causing cells to be more resistant to oxidative stress [43].

Interestingly, pharmacological inhibition of PIK3CA kinase activity with alpelisib sensitized KP cells to ROS but not to the same degree as complete ablation of the protein. Additionally, the drug did not affect the metabolic response to glutathione depletion. This suggests that there may exist some additional mechanism affecting metabolic signaling in KPA cells beyond the kinase activity of PIK3CA, including possibly a structural role of PIK3CA itself. While PIK3CA is predominantly known for its kinase-related functions, it also structurally contains domains for direct RAS binding, two SH2 domains, and a helical domain where it interacts with the p85 regulatory subunit of PI3K [72]. Disruption of the RBD is established to impede cell growth and proliferation [7], but since ablation of kinase activity is not phenotypically synonymous with deletion of the PIK3CA protein, it is possible that PIK3CA may have additional non-enzymatic functions that affect cellular redox, possibly including its interactions with p85. Therefore, future studies are required to further investigate potential non-kinase and non-KRAS binding roles for PIK3CA in tumor cell growth *in vitro* and *in vivo*.

Furthermore, KPA and KPS cells both demonstrated significant resistance to first-line NSCLC chemotherapies which aligns with the clinically established chemotherapy-resistant nature of KRAS-mutant NSCLC with loss of STK11 function [73]. The increased resistance of KPA suggests that complete inhibition of the PI3K pathway in KRAS-mutant NSCLC with wild-type PIK3CA may induce resistance to traditional cytotoxic chemotherapy, possibly due to decreased mitotic rates. However, the lack of *in vivo* growth of KPA cells with loss of PIK3CA suggests a contradictory effect. Future studies using KPA cells could investigate these two distinct facets of PIK3CA in KRAS-mutant NSCLC.

Several orthotopic implantation models for NSCLC have previously been described [74,75]. However, these have involved the intrathoracic implantation of a xenograft [74] or used intratracheal delivery of organoids or cells, which requires bleomycin pre-treatment for successful cell delivery [75]. Therefore, our model benefits from the use of syngeneic cells directly implanted into the lung without anti-mitotic chemotherapy treatment for the least disruption to the host lung microenvironment. Through the implementation and characterization of this model, we have demonstrated its utility with the implantation of our cell line lacking PIK3CA having the novel ability to grow *in vitro* but not *in vivo*, while our KP and KPS cell lines exhibited similar *in vitro* and *in vivo* growth. Our preliminary studies suggest a sensitivity of the KPA cell line to ROS in comparison to parental KP or KPS cells that may affect tumor development in the lung but is not encountered in standard cell culture conditions. One potential difference in the *in vitro* environment could relate to the aerobic composition of the microenvironment in a lung. However, the ratio of partial pressure of oxygen between a tumor and its surrounding lung tissue has been measured to have high variability between human NSCLC patients [76], making this a potentially complicated feature to mimic. Some cell culture strategies, including air–liquid interface [77] and oxygen-permeable membranes with a hypoxic chamber [78], have been described by others to more closely simulate physiologically relevant lung tumor conditions. Future studies could also look more closely at the development of microscopic KPA tumors following implantation, rather than focusing on visible macroscopic tumors. Further work is necessary to understand the mechanism for the lack of KPA tumor development *in vivo*, and this model provides a unique opportunity to study the effects of PIK3CA ablation in KRAS-driven LUAD.

## 5. Conclusions

We have determined that loss of PIK3CA from KRAS-mutant lung adenocarcinoma prevents the continued growth and progression of tumors *in vivo*, even when implanted directly into the lungs of immunodeficient mice, while markedly increasing sensitivity *in vitro* to oxidative stress from hydrogen peroxide. This provides a model to better understand the fundamental requirements for tumor growth *in vivo* and thus potentially identify new therapeutic approaches.

## Data Availability

The RNA-sequencing raw and processed data has been deposited into the Gene Expression Omnibus (GEO) under accession number GSE306343.

## Supplementary Materials

The following supporting information can be downloaded at: https://www.mdpi.com/article/10.3390/cells15060506/s1, Figure S1: *In vivo* LUAD model with KP cell line. 5 × 10^5^ KP cells were implanted into the right (*n* = 8) or left (*n* = 6) lung of mice. The cell solution injected into the left lung also contained 500 μg/mL growth factor-reduced Matrigel. (A) Tumor progression was observed with IVIS imaging at weekly intervals points post-implantation. Images from one representative mouse are shown. Scale represents luminescence intensity (arbitrary units). (B) Kaplan-Meier survival curves showing no significant difference and a median survival time of 22 days for both groups. (C) Macroscopic image of chest cavity from one mouse that died of tumor progression. Scale bar represents 10 mm. (D) H&E staining of lung section showing tumor (T) adjacent to normal lung tissue (L). Scale bar represents 200 μm; Figure S2: Lack of KPA *in vivo* tumor progression is not due to adaptive immune response or seeding density. (A) Kaplan-Meier survival curves for B6 or SCID mice implanted with 5 × 10^5^ KPA cells or KP cells as control in lung (*n* = 6 per group). Median survival: B6 + KP, 38 days; all mice injected with KPA cells were alive on day 82. *p* < 0.009, KP versus KPA-injected mice (log-rank test). (B) To test whether simply increasing the number of initial KPA cells may overcome growth limitations, B6 (*n* = 6) and SCID mice (*n* = 5) were implanted with ten-fold more (5 × 10^6^) KPA cells. All B6 and SCID mice were alive on day 82 suggesting growth limitations cannot be overcome simply by increased cell numbers.
